# Effects of orthogonal dual-frequency ultrasound-assisted treatment combined with bioactive coating containing *Melissa officinalis* L. essential oil on changes in quality, lipid, and protein of large yellow croaker (*Pseudosciaena crocea*) during cold storage

**DOI:** 10.1016/j.fochx.2024.101861

**Published:** 2024-09-27

**Authors:** Hao Cheng, Chenchen Zhang, Jinfeng Wang, Jing Xie

**Affiliations:** a College of Food Science and Technology, Shanghai Ocean University, Shanghai, China; b Key Laboratory of Aquatic Products High Quality Utilization, Storage and Transportation (Co-construction by Ministry and Province), Ministry of Agriculture and Rural Affairs, Shanghai, China; c National Experimental Teaching Demonstration Center for Food Science and Engineering, Shanghai Ocean University, Shanghai, China; d Shanghai Engineering Research Center of Aquatic Product Processing and Preservation, Shanghai, China; e Shanghai Professional Technology Service Platform on Cold Chain Equipment Performance and Energy Saving Evaluation, Shanghai, China

**Keywords:** Cold storage, Large yellow croaker, Orthogonal dual-frequency ultrasound-assisted, Bioactive coating, *Melissa officinalis* L. essential oil, Quality attributes

## Abstract

How to reduce the quality loss of aquatic products during storage is a topic worth exploring. This study proposed a method combining orthogonal dual-frequency ultrasound-assisted treatment (20 kHz vertically, 40 kHz horizontally, 400 W) with bioactive coating (*Melissa officinalis* L. essential oil-carboxymethyl chitosan-locust bean gum) and discussed the effects of this combined treatment on the quality, lipid, and protein of large yellow croaker during cold storage (4 °C). The results showed that both ultrasound-assisted treatment (US) and bioactive coating (CMCS) significantly inhibited microbial growth and quality deterioration in the fish, with the combined treatment group (US+CMCS) showing the best effect. The shelf life of large yellow croaker in the control group (CK) was 6 d, while the shelf life for US, CMCS, and US+CMCS treatments was 12 d, 12 d, and 18 d, respectively. Additionally, the combined treatment inhibited lipid oxidation and effectively delayed the oxidative degradation of protein in the large yellow croaker during cold storage. Therefore, the method of orthogonal dual-frequency ultrasound-assisted treatment (20 kHz vertically, 40 kHz horizontally, 400 W) combined with bioactive coating (*Melissa officinalis* L. essential oil-carboxymethyl chitosan-locust bean gum) proposed in this study was a promising approach for the preservation of aquatic products during storage.

## Highlights

• A novel orthogonal dual-frequency ultrasound-assisted treatment was provided.
• Bioactive coating with added *Melissa officinalis* L. essential oil was produced.
• Combined treatment of ultrasound-assisted and bioactive coatings was performed.
• Combined treatment could delay the quality deterioration of refrigerated fish.

## Graphical abstract

**Figure:**
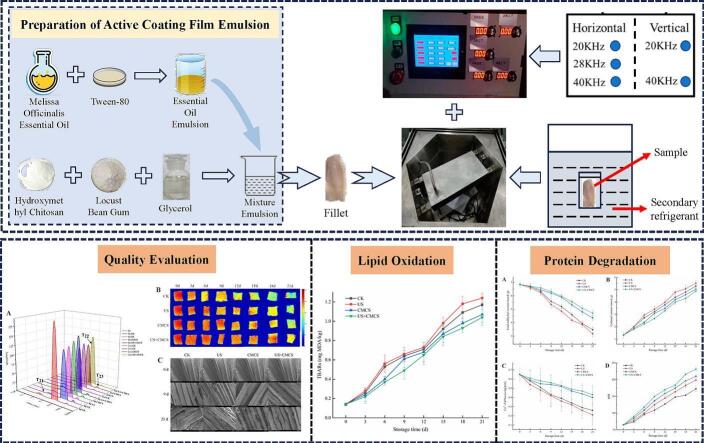

## 1 Introduction

Large yellow croaker (*Pseudosciaena crocea*) is a species of fish that holds significant economic and cultural value in East Asia, particularly in China (Cheng et al., 2022). Renowned for its delicious taste and high nutritional value, over the years, the large yellow croaker has become an important subject of aquaculture (J. Wang et al., 2024). However, during transportation and storage, the quality of large yellow croaker after leaving the water gradually deteriorates due to environmental factors and biochemical reactions (Mao et al., 2024). This significantly reduces its usability in production and processing, as well as consumer acceptance. Therefore, finding effective methods to slow down and mitigate the quality deterioration of large yellow croaker during transportation and storage has become a topic of considerable interest and discussion.

**Fig. 1:**
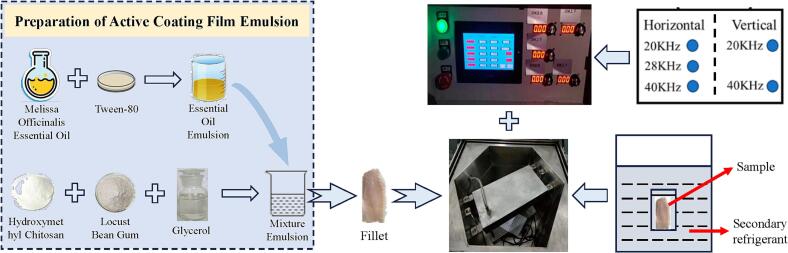
Schematic diagram of the experimental design and treatment.

Ultrasound-assisted technology has emerged as a promising method in various fields, including food processing, medicine, and environmental science (Zheng et al., 2024). This innovative technology utilizes high-frequency sound waves to enhance physical, chemical, and biological processes. In the food industry, ultrasound-assisted technology is particularly valued for its ability to improve extraction efficiency, enhance preservation methods, and reduce processing times (Kumar et al., 2023). By creating microscopic cavitation bubbles, the technology can disrupt cell walls, increase mass transfer, and promote chemical reactions, leading to improved product quality and shelf life (S. Zhou et al., 2024). However, ultrasound-assisted technology has not yet been applied to the refrigerated processing of aquatic products. Based on these, we have developed a device suitable for the pretreatment of large yellow croaker. This device consists of two main components: an ultrasonic equipment tank and a controller (as briefly shown in Fig. 1). The controller can process the samples in the ultrasonic equipment tank by adjusting different frequencies both vertically (20 kHz and 40 kHz) and horizontally (20 kHz, 28 kHz, and 40 kHz).

On the other hand, the application of bioactive coating has become increasingly widespread, primarily in food storage and preservation. These bioactive coatings, which include various plant-derived bioactive substances, can impart additional biological properties, such as antibacterial and antioxidant functions (Díaz-Montes & Castro-Muñoz, 2021). Hydroxypropyl chitosan, a natural polysaccharide, is used as a base material for functional films due to its antibacterial properties, biodegradability, good water solubility, and excellent film-forming characteristics (Kulawik et al., 2024). Locust bean gum, a natural polymer based on polysaccharides, has flexibility that allows it to form hydrogen bonds with water molecules, a key property for biomaterials, and its film-forming performance is crucial for food packaging applications (Sana et al., 2024). Therefore, hydroxypropyl chitosan and locust bean gum as film matrices have broad application potential in the packaging industry. *Melissa officinalis* L. essential oil, recognized as safe within standard regulations, contains many bioactive compounds, among which rosmarinic acid is particularly important (Carvalho et al., 2021). It has reliable antibacterial and antioxidant capabilities, making it a valuable option for incorporation into food for preservation purposes. The research conducted by Simat et al. (Šimat et al., 2023) demonstrated that the addition of *Melissa officinalis* L extract did not negatively affect the sensory properties of shrimp; on the contrary, it was observed that the extract addition group had the highest overall sensory scores and was able to prolong the shelf life of cold brined shrimp from 4 months to 8 months. There are also more reports on the application of *Melissa officinalis* L as an extract for product preservation, all of which have shown positive results (Carvalho et al., 2021; Pogorzelska-Nowicka et al., 2024; Shetta et al., 2024).

Therefore, this study evaluated the effects of ultrasound-assisted treatment combined with *Melissa officinalis* L. essential oil-carboxymethyl chitosan-locust bean gum bioactive coating on the quality, lipid, and protein characteristics of large yellow croaker during cold storage. The main analyses including water holding capacity (WHC), pH, total volatile basic nitrogen (TVB-N), K value, microorganism, moisture distribution and migration, microstructure, thiobarbituric acid reactive substances (TBARs), and myofibrillar protein (MP) characteristics were conducted.

## 2 Materials and methods

### 2.1 Raw material and sampling

A total of 58 large yellow croaker from the same batch were used in this study, with 2 fish designated for freshness experiment. The raw materials were purchased from the nearby Luchaogang Seafood Market (Shanghai), with an average weight of 500 g per fish. Before being transported to the laboratory, they were kept alive in oxygenated bags. Upon arrival at the laboratory, the fish were stunned by a blow to the head, followed by the rapid removal of the head, viscera, and skin. Each fish was then divided into two fillets of similar shape and size. The fillets were thoroughly washed with a 1 % sodium chloride solution and patted dry to remove surface moisture. The prepared fillets were collected and set aside for further experiment. All handling procedures adhered to relevant ethical regulations as outlined in EU Directive 2010/63/EU for animal experiments (application number SHOU-DW-2023-50 for ethical review of animal experiments in our university).

### 2.2 Preparation of coating composed of *Melissa officinalis* L. essential oil, carboxymethyl chitosan, and locust bean gum

The preparation of the *Melissa officinalis* L. essential oil emulsion was based on the method described by Ghosh et al. (Ghosh et al., 2014). Tween-80 was used as an emulsifier. *Melissa officinalis* L. essential oil (10 % *v*/v) and Tween-80 were added to deionized water. The coarse emulsion was then processed using a sonicator with a 13 mm diameter probe at 20 kHz and 750 W in pulse mode (30 s on and 30 s off) for 15 min to obtain the desired *Melissa officinalis* L. essential oil emulsion.

The preparation of the carboxymethyl chitosan-locust bean gum bioactive coating was based on the method described by Keawpeng et al. (Keawpeng et al., 2022). 1 g of carboxymethyl chitosan and 1 g of locust bean gum were added to 200 mL of deionized water. 0.4 g of glycerol was added as a plasticizer, and the mixture was stirred and heated at 60 °C until the polymers were completely dissolved. When the mixture cooled to 35 °C, the previously prepared *Melissa officinalis* L. essential oil emulsion was added to obtain the coating solution required for the experiment. The final concentration of *Melissa officinalis* L. essential oil was 1 μg/mL.

### 2.3 Procedure for ultrasound-assisted treatment

Before the experiment, the ultrasound equipment (Xiecheng Ultrasonic Equipment Co., Ltd., Jining, Shandong, China) was turned on 2 h in advance to bring the secondary refrigerant down to 4 °C. During ultrasound-assisted treatment, the samples were placed in the ultrasound device and treated at the same frequency and power (20 kHz vertically, 40 kHz horizontally, 400 W) with a cycle of 30 s on and 30 s off for 30 min.

The treated fillets were individually packaged in sterile vacuum-sealed bags. One group received no treatment and was set as the CK group; one group underwent only ultrasound-assisted treatment and was set as the US group; one group received only the coating treatment and was set as the CMCS group; and one group received both ultrasound-assisted and coating treatments and was set as the US+CMCS group. The treated samples were stored in a refrigerator at 4 °C, and random samples were taken for analysis on 0 d, 3 d, 6 d, 9 d, 12 d, 15 d, 18 d, and 21 d. The detailed treatment process was displayed in Fig. 1.

### 2.4 Determination of quality evaluation

#### 2.4.1 WHC

2 g of samples were collected for centrifugation at 5, 000 ×*g* for 10 min. A triple of each measurement was taken. And WHC was calculated using the following equation:

(1) $$\mathrm{WHC}\;\left(\%\right)=\frac{W_{2}}{W_{1}}\times 100\%\#$$

where *W*_*1*_ referred to the weight before centrifugation, and *W*_*2*_ referred to the weight after centrifugation (Cheng et al., 2022).

#### 2.4.2 pH

In this experiment, 2 g of samples were mixed with 18 mL of physiological saline solution (0.9 % m/v) and centrifuged at 1, 000 ×*g* for 5 min, and the pH was measured in triplicate (Cheng et al., 2023).

#### 2.4.3 TVB-N

A mixture of 5 g samples and MgO was determined with a Kjeltec 8400 nitrogen analyzer (Denmark FOSS China Shanghai Co., Ltd.). Each measurement was made three times (Cheng et al., 2024).

#### 2.4.4 K value

The K value was determined using a freshness meter (QS-3201, QS-SOLUTION, Tokyo, Japan). A 0.3 g sample of large yellow croaker was cut and added to 600 mL of extraction solution. The fish meat was cut with scissors for 30 to 60 s. To neutralize the sample's pH, pH paper was used to ensure the solution was neutral, with a pH value between 6 and 8. After standing, the supernatant was collected for electrophoretic separation. The freshness of the sample was then rapidly analyzed and calculated using image analysis to determine the K value.

#### 2.4.5 microorganism

Microbial analysis was performed following the method of Liu et al. (W. Liu et al., 2021) to determine the total viable count and psychrophilic bacteria. A 5 g sample of large yellow croaker meat was added to 45 mL of sterile physiological saline and homogenized using a stomacher. The homogenized solution was then diluted with sterile physiological saline at a ratio of 1:10 (*v*/v). A 100 μL aliquot of the homogenized solution was plated on plate count agar medium. The plates were incubated at 37 °C for 2 d and at 4 °C for 7 d to record the total viable count and psychrophilic bacteria count, respectively. Each count was expressed as the logarithm of the colony-forming unit (lg CFU/g).

#### 2.4.6 Water distribution and migration

The water distribution and migration in the fishes were evaluated via a low-field nuclear magnetic resonance (LF-NMR) analyzer (MesoMR23–060H·I, NiuMeng, Shanghai, China) on samples measuring 2 cm × 2 cm × 1 cm. The Niumeng Electric Corporation's MultiExp Inv Analysis program was used to analyze the data from 5, 000 echoes of 8 scan repetitions. Each test was run at least three times (Yang et al., 2024).

#### 2.4.7 Microstructure

The large yellow croaker samples were cut into slices of 3 mm × 3 mm × 1.5 mm using a Leica microtome. The slices were then fixed in 2.5 % glutaraldehyde solution at 4 °C for 24 h. After fixation, the solution was discarded, and the samples were rinsed three times with 0.1 mol/L phosphate buffer (pH = 7.3), each rinse lasting 15 min. The samples were then subjected to a graded ethanol series (30 %, 50 %, 70 %, 80 %, 90 %, 95 %, and 100 %) for dehydration, followed by substitution of ethanol with isoamyl acetate. Finally, the treated slices were freeze-dried in a freeze-dryer (FDU-2110, AC 220 V, 50 Hz, 2.4 KVA, maximum working power is 11 A) for 3 d. After freeze-drying, the samples were sputter-coated with gold in an ion sputter coater and observed using a scanning electron microscope (SU 5000, HITACHI, Japan) at an accelerating voltage of 20 kV (Cheng et al., 2024).

### 2.5 Determination of TBARs

Accurately weigh 5 g of fish meat and mechanically homogenize it. 25 mL of 20 % trichloroacetic acid was added to the homogenized mixture and allowed to stand for 1 h. The mixture was centrifuged at 0 °C and 8, 000 ×g for 10 min. The filtrate was transferred to a 50 mL volumetric flask after filtration. Using a pipette, transfer 5 mL of the above solution into a beaker. 5 ml of 0.02 mol/L TBA reagent was added and mixed thoroughly. The mixture was reacted in boiling water for 20 min, then removed and cooled to room temperature. The absorbance was measured at 532 nm using distilled water as the blank. The TBARs value was calculated using the following formula:

(2) $$\begin{array}{c} \text{TBARs}=0.78\;A \end{array}$$

where TBARs was expressed in mg/kg, and the A was the absorbance value at 532 nm (R. Zhou et al., 2011).

### 2.6 Extraction of MP

With slight modifications, the extraction of MP was performed following the method described by Xu et al. (Ni et al., 2024). To homogenize 2 g of samples, 20 mL of 20 mmol/L Tris-HCl buffer was used. The mixture was centrifuged at 10, 000 ×*g* for 15 min. The precipitate was collected, and then an additional 20 mL of 20 mmol/L Tris-HCl buffer was added and stirred for 3 h. The centrifugation step at 10, 000 ×*g* for 15 min was repeated, and the supernatant was used to extract the MP.

### 2.7 Determination of MP

#### 2.7.1 Total sulfhydryl content, carbonyl content, and Ca^2+^-ATPase activity

The method outlined in the test kit (Beijing Solarbio Science & Technology Co., Ltd) was used to determine the total sulfhydryl content, carbonyl content, and Ca^2+^-ATPase activity. The results were displayed as μmol/g pro, μmol/g pro, and U/mg pro, respectively.

#### 2.7.2 Myofibrillar fragmentation index (MFI)

For the measurement of MFI, the protein was diluted to a concentration of 0.5 mg/mL. The absorbance of the diluted solution was measured at 540 nm using a UV spectrophotometer. Finally, the MFI value was calculated by multiplying the absorbance by 200 (Kara et al., 2024).

### 2.8 Statistical analysis

The data was analyzed using SPSS 23.0 statistical software, graphs were generated via Origin 2018.

## 3 Results and discussion

### 3.1 Evaluation of quality

#### 3.1.1 WHC

**Fig. 2:**
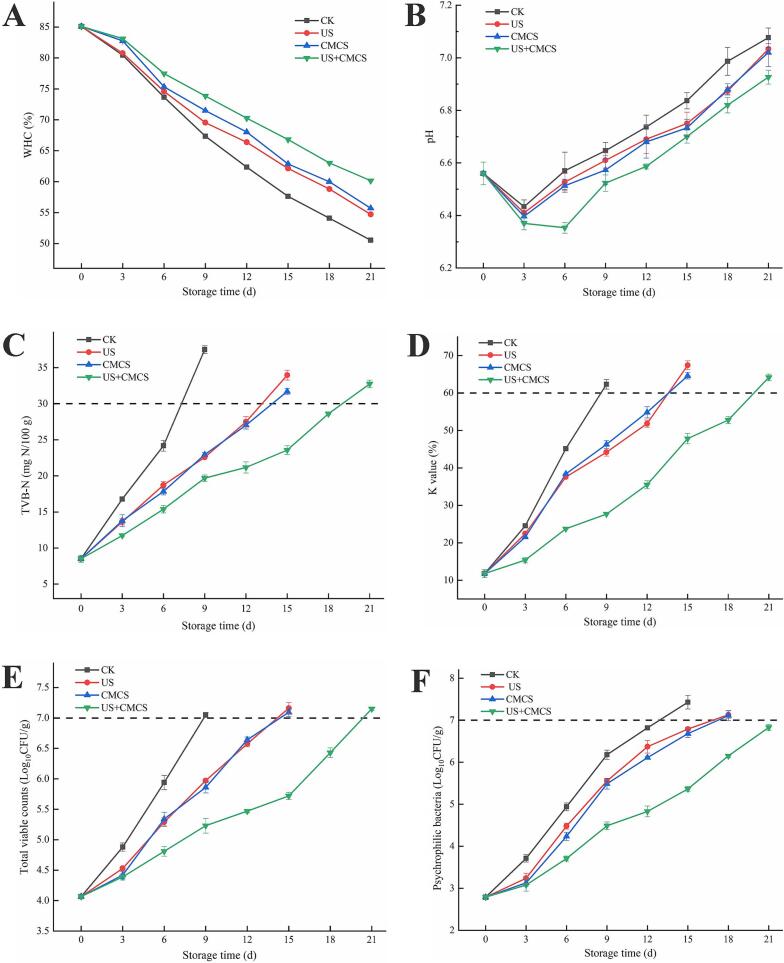
Changes in WHC (A), pH (B), TVB-N (C), K value (D), total viable counts (E), and psychrophilic bacteria (F) of large yellow croaker with different treatments during cold storage. (For interpretation of the references to colour in this figure legend, the reader is referred to the web version of this article.)

WHC is an important quality parameter that affects the appearance and storage stability of aquatic products. As shown in Fig. 2A, the WHC of fresh large yellow croaker was 85.09 %, and the WHC of all groups decreased with extended storage time. This decline was mainly due to the instability of fish muscle protein-water interactions, degradation of myofibrillar proteins, and bacterial growth, which enlarged the extracellular gaps and affected the WHC of the fish (Cheng et al., 2022). During storage, the WHC of both CK group and US group significantly decreased, becoming lower than that of CMCS and US + CMCS after 6 d. Based on the total viable count and psychrophilic bacteria test results, it was hypothesized that the increase in microbial load within the fish during storage accelerated protein degradation and water loss. From the 9th day onward, the WHC of the US + CMCS group was significantly better than that of other groups. Specifically, the WHC of the CK group dropped to 57.63 % by the 15th day, whereas the US + CMCS group maintained a WHC of 66.82 %. This indicated that the combination of ultrasound-assisted and CMCS could sustain the moisture stability of large yellow croaker.

#### 3.1.2 pH

As shown in Fig. 2B, the pH of large yellow croaker during storage under different treatments exhibited a “V” shape, decreasing from 0 d to 3 d and increasing from 6 d to the end of storage. Sun et al. (Sun, Guo, et al., 2019) also observed a similar trend in grass carp wrapped in a fish gelatin film containing curcumin/β-cyclodextrin emulsion. The initial decrease in pH was primarily due to glycolysis of glycogen, producing lactic acid, the release of inorganic phosphate, and the accumulation of lactic acid during the initial rigor mortis phase of the seafood. The subsequent increase in pH was associated with fish spoilage caused by microbial activity (Mahmoud et al., 2006). Microorganisms utilized amino acids in the fish, producing alkaline substances such as ammonia, trimethylamine, or other basic nitrogen compounds, raising the pH. Samples treated with *Melissa officinalis* L. essential oil coating showed a lower pH than the CK group, particularly on 9 d and 15 d. The antibacterial properties of *Melissa officinalis* L. essential oil and carboxymethyl chitosan likely contributed to the lower pH in these treated samples, as inhibiting spoilage bacteria reduced the production of alkaline substances (X. P. Li et al., 2017; Yu et al., 2018). At the end of storage, the lower pH of the samples indicated a lower degree of spoilage. After 15 days of storage, the pH values of all groups continued to increase, and the CK group was higher than the other groups, while the CMCS group showed a lower trend. In the early storage period, the pH of the US group was higher than that of the CMCS group, possibly because ultrasound-assisted treatment delayed the rigor mortis phase, causing a rise in pH (Got et al., 1999). The pH of the CMCS and US + CMCS groups remained consistently low, with the US + CMCS group maintaining the lowest level at the end of storage, which was related to the acidity of *Melissa officinalis* L. essential oil. The combined treatment effectively inhibited bacterial growth, delayed protein degradation, and maintained a low pH.

#### 3.1.3 TVB-N

TVB-N refers to basic nitrogenous compounds such as ammonia and amines produced by the degradation of animal-derived foods by bacteria or enzymes. It is an important indicator for measuring the freshness of fish (Olatunde et al., 2019). As shown in Fig. 2C, the TVB-N of fresh large yellow croaker on 0 d was 8.52 mg N/100 g. The TVB-N of the CK group samples increased significantly with storage time and was noticeably higher than that of the other groups, indicating that the control group samples had quickly entered a state of spoilage, reaching 37.51 mg N/100 g on 9 d, which was beyond the spoilage threshold. The TVB-N of the US group and CMCS group samples exceeded the secondary freshness level on 15 d, reaching 34.00 mg N/100 g and 31.69 mg N/100 g, respectively. However, the US + CMCS samples didn't exceed the spoilage limit until 21 d, with TVB-N of 32.74 mg N/100 g. The cavitation effect of ultrasound could alter the structure of microorganisms, leading to their death, thereby preventing protein degradation and reducing the formation of basic nitrogen compounds such as ammonia and amines. Additionally, CMCS has some antibacterial effects, and under acidic conditions, enzyme activity is inhibited, slowing down protein degradation and the rise in TVB-N (Ni et al., 2024). Therefore, the rate of increase in TVB-N in the combined treatment group samples was slower than that in the other treatment groups, which corresponding to the microbial indicators.

#### 3.1.4 K value

A higher K value indicates poorer freshness, with the acceptable upper limit being 60 % (Ocaño-Higuera et al., 2011). The K value of fresh large yellow croaker was 11.79 % (Fig. 2D). The initial increase in the K value was due to the decomposition of ATP and its derivatives, while the subsequent increase was due to microbial activity (Zhao et al., 2021). The CK group exceeded the upper limit on 9 d, reaching 62.99 %. The US and CMCS group samples exceeded the upper limit on 15 d, with values of 67.41 % and 64.61 %, respectively. By the end of storage, the K value in the US + CMCS group also exceeded the upper limit. This indicated that both ultrasound treatment and *Melissa officinalis* L. essential oil could delay the degradation of IMP and the production of HxR and Hx by inhibiting microbial growth, resulting in a lower K value. Therefore, the rate of increase in the K value in the samples treated with the combined ultrasound-assisted and *Melissa officinalis* L. essential oil coating was significantly slower than that in the other groups. After the death of fish, ATP began to be degraded and IMP accumulated consequently. However, with the extension of storage time, IMP was further degraded to HxR and Hx, and the accumulation of both was the key factor leading to the increase of K value. Degradation of nucleotides is the main process affecting the K-value and is usually catalyzed by enzymes (e.g., ATPase, AMPase, IMPase, etc.). The high-frequency vibration and cavitation effects of ultrasound may disrupt the structure or activity of these enzymes, thereby inhibiting the breakdown of nucleotides and reducing the rate of conversion of IMP to HxR and Hx. This will retard the rise in K value and maintain the freshness of the fish. On the other hand, essential oils have broad-spectrum antimicrobial activity, which can effectively inhibit the growth of spoilage microorganisms that lead to the decline of fish freshness. The metabolic activity of microorganisms accelerates the degradation of ATP, leading to a rise in K-value. By inhibiting the growth of microorganisms, essential oils can slow down the decomposition of nucleotides, thus delaying the rise of K value.

#### 3.1.5 Total viable counts and psychrophilic bacteria

Fig. 2E-F showed the microbial changes in large yellow croaker samples during cold storage. The total viable count of fresh large yellow croaker was 4.07 lg CFU/g (Fig. 2E), indicating a good quality. During storage, the total viable count increased in all large yellow croaker samples, with those treated with *Melissa officinalis* L. essential oil coating and ultrasound-assisted having lower counts than untreated samples. The untreated group had the highest total viable count, exceeding the acceptable maximum limit for marine fish of 7 lg CFU/g on 9 d. The total viable count of the ultrasound-assisted, *Melissa officinalis* L. essential oil-coated, and combined ultrasound-assisted and *Melissa officinalis* L. essential oil-coated samples exceeded the maximum limit on 12 d, 12 d, and 21 d, respectively. On 18 d, the total viable count in the US + CMCS group was 6.43 lg CFU/g, still below the maximum limit. This indicated that ultrasound treatment and the addition of *Melissa officinalis* L. essential oil to the bioactive coating could inhibit microbial growth.

As shown in Fig. 2F, the psychrotrophic bacterial count was 2.79 lg CFU/g on 0 d and gradually increased during cold storage. Compared to the CK group, the ultrasound-assisted group and the *Melissa officinalis* L. essential oil-coated group had lower psychrotrophic bacterial growth. Both ultrasound treatment and *Melissa officinalis* L. essential oil significantly inhibited the growth of psychrotrophic bacteria (Vieira et al., 2019). The US + CMCS group showed the slowest growth of psychrotrophic bacteria, followed by the CMCS group. On 15 d, the psychrotrophic bacterial counts in the CMCS and US + CMCS groups were 6.68 and 5.37 lg CFU/g, respectively, while the counts in the CK and US samples were 7.43 and 6.79 lg CFU/g, respectively.

The bioactive coating containing *Melissa officinalis* L. essential oil significantly delayed microbial growth in large yellow croaker during cold storage. The total viable count and psychrotrophic bacterial count in the US group also increased more slowly. Bonilla et al. (Bonilla et al., 2013) explained that bioactive coating had oxygen barrier properties, which to some extent slow down microbial growth during cold storage of large yellow croaker. The US + CMCS group showed the best antibacterial effect, indicating a synergistic effect between ultrasound-assisted and *Melissa officinalis* L. essential oil. Due to the cavitation effect of ultrasound, the *Melissa officinalis* L. essential oil could penetrate into the muscle of large yellow croaker, enhancing its antibacterial effect.

### 3.2 Water distribution and migration

**Fig. 3:**
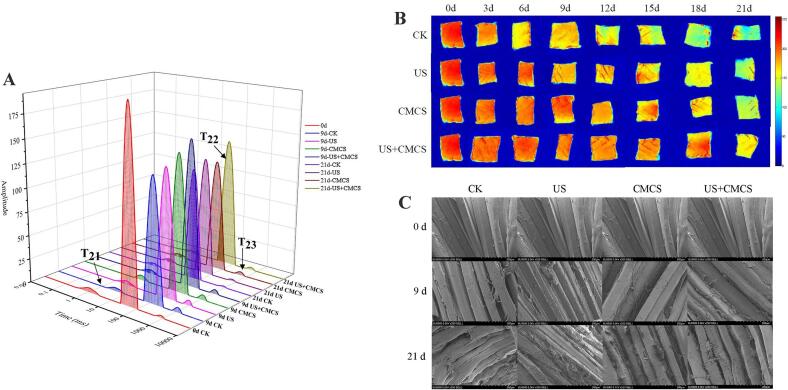
Changes in water distribution (A), magnetic resonance imaging (B), and microstructure (C) of large yellow croaker with different treatments during cold storage. (For interpretation of the references to colour in this figure legend, the reader is referred to the web version of this article.)

The LF-NMR technique analyzes the transverse relaxation time T_2_ of large yellow croaker to determine the moisture distribution and migration during cold storage, thereby revealing the moisture migration patterns during cold storage. T_2_ includes T_21_ (< 10 ms), T_22_ (10 ms < T_22_ < 100 ms), and T_23_ (> 100 ms), which represent bound water, immobilized water, and free water, respectively (Cheng et al., 2022). As shown in Fig. 3A, the T_21_ peak area of each component didn't change significantly during storage, indicating that the bound water among the components remained relatively stable. The T_22_ of the US group remained stable and higher than that of the CK group, indicating that ultrasound-assisted treatment positively affected the water holding capacity of large yellow croaker. In the early storage period, the T_22_ of the CMCS and US + CMCS group samples was significantly lower than that of the control group. This was because *Melissa officinalis* L. essential oil coating damaged the connective tissue and cell membranes, leading to decreased water holding capacity. Additionally, the acidic nature of *Melissa officinalis* L. essential oil coating could cause protein denaturation and changes in the chemical equilibrium between protein-protein and protein-water at low pH, leading to increased water release (Y. Liu et al., 2023). In the later storage period, the T_22_ of the CMCS and US + CMCS groups remained stable, especially the US + CMCS group, which experienced less moisture loss. This was due to the excellent antibacterial activity of *Melissa officinalis* L. essential oil coating, which delayed muscle protein degradation and protected the structure of myofibrils. Ultrasound exposed the hydrophilic groups in proteins, reducing water mobility and making it difficult for water molecules to migrate.

MRI visually displays the morphological structure, internal structure, and molecular distribution of large yellow croaker. High proton concentration appears as high-humidity red, and low proton concentration appears as low-humidity blue (Lin et al., 2019). As shown in Fig. 3B, the colour of the samples in each group changed from red to yellow with increasing storage time. The CK group exhibited the most significant change, turning bright yellow by 6 d, indicating substantial moisture loss. During this period, the US + CMCS samples remained red, while the other three groups tended toward red. After 15 d, the US + CMCS samples still appeared orange-red, with slow water loss, indicating that the US + CMCS combination significantly maintained moisture, consistent with the results of LF-NMR.

### 3.3 Microstructure

As shown in Fig. 3C, the images displayed the large yellow croaker samples under different treatments at 250× magnification using a scanning electron microscope (SEM) during cold storage. It could be observed that, as storage time increased, the myofibril arrangement in all groups became disordered, the distance between muscle bundles widened, the muscle structure loosened, and cell rupture even occurred. From the electron microscope images of the US + CMCS group samples on 9 d, it was evident that a small amount of *Melissa officinalis* L. essential oil coating had entered the myofibril gaps. This phenomenon was due to the cavitation and mechanical effects of ultrasound. On 21 d, the myofibril structure of the US + CMCS group remained the best and showed the least change compared to the other three groups. Therefore, it could be concluded that the combined treatment of ultrasound-assisted and *Melissa officinalis* L. essential oil coating significantly slowed down the structural changes in myofibrils compared to the other groups.

### 3.4 Analysis of TBARs

**Fig. 4:**
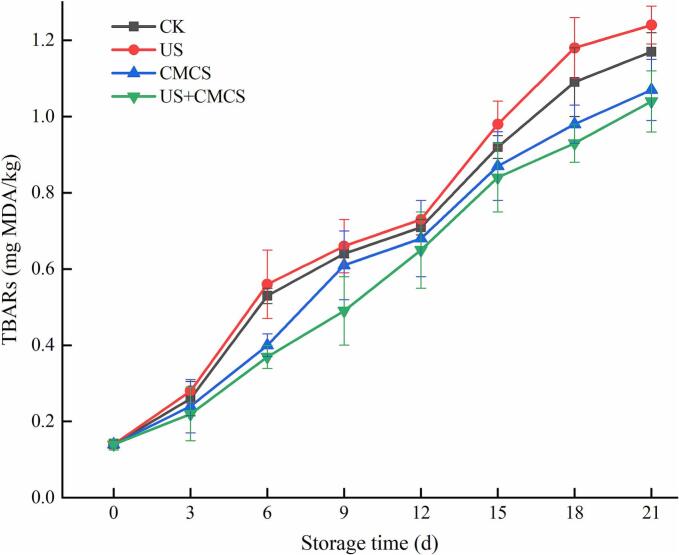
Changes in TBARs of large yellow croaker with different treatments during cold storage. (For interpretation of the references to colour in this figure legend, the reader is referred to the web version of this article.)

According to Fig. 4, the TBARs in all groups showed an overall increasing trend with extended storage time. During storage, the TBARs of the US group were higher than those of the CK group, possibly due to the free radical oxidation of lipids caused by reactive oxygen species generated by ultrasound (Chaijan et al., 2020). In contrast, the TBARs of the CMCS and US + CMCS groups were significantly lower than those of the CK and US groups throughout the storage period, indicating that CMCS effectively inhibited lipid oxidation. This might be because the phenolic compounds in *Melissa officinalis* L. essential oil could slow down the lipid oxidation process and scavenge free radicals, thus inhibiting lipid oxidation reactions. The antioxidant activity of *Melissa officinalis* L. essential oil, combined with the antioxidant properties of the bioactive coating, could enhance the inhibition of lipid oxidation by reducing free radical formation and subsequently preventing the oxidation of myofibrils (Sun, Sun, et al., 2019Furthermore, the TBARs in the US + CMCS group were significantly lower than those in the other groups during storage, indicating that the combination of ultrasound and *Melissa officinalis* L. essential oil could effectively inhibit lipid oxidation (Zhan et al., 2018).

### 3.5 Analysis of MP

#### 3.5.1 Total sulfhydryl content

**Fig. 5:**
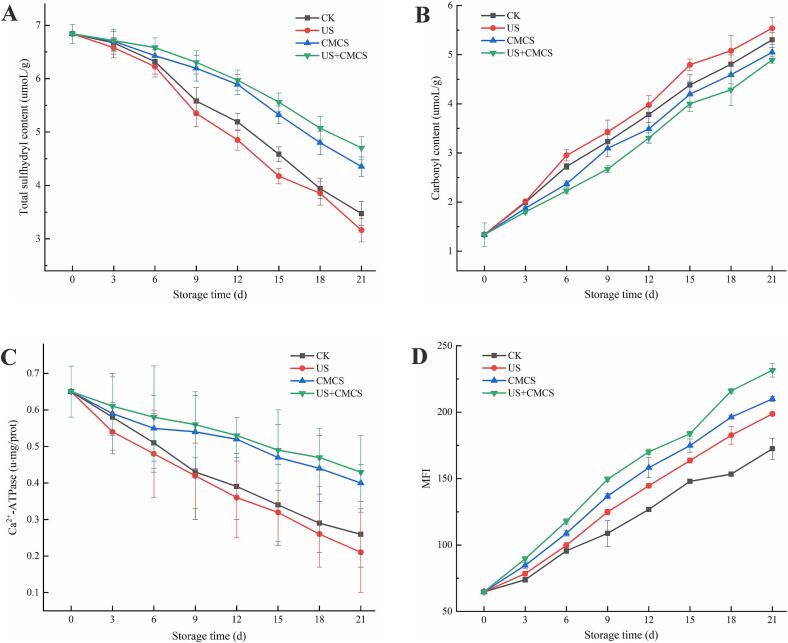
Changes in total sulfhydryl content (A), carbonyl content (B), Ca^2+^-ATPase (C), and MFI (D) of large yellow croaker with different treatments during cold storage. (For interpretation of the references to colour in this figure legend, the reader is referred to the web version of this article.)

Sulfhydryl, as key functional groups in protein amino acid residues, can be used to evaluate their oxidation levels (B. Wang et al., 2020). A decrease in total sulfhydryl content indicates an increase in protein oxidation, while a higher sulfhydryl content suggests lower oxidation levels of MP (*P.* Gao et al., 2009). According to Fig. 5A, the sulfhydryl content in all treatment groups showed a decreasing trend over time, indicating that MP oxidation had occurred. Among the different treatment groups, the sulfhydryl content was lower after ultrasound treatment due to the mechanical vibrations and impact effects of ultrasound, which generate shear forces that alter the intermolecular interactions and structure within the sample. This might lead to changes in the binding of sulfhydryl molecules with surrounding molecules, reducing their content. The sulfhydryl content in the US + CMCS group was the highest, indicating that the membrane solution containing *Melissa officinalis* L. essential oil significantly slowed down the reduction rate of sulfhydryl content, suggesting that the active coating can delay the oxidation of myofibrils in large yellow croaker. Study has reported that natural extracts containing phenolic compounds can preferentially react with free radicals, preventing thiol oxidation (Mei et al., 2013). *Melissa officinalis* L. essential oil competes with sulfhydryl to capture free radicals, thus inhibiting sulfhydryl oxidation (Z. Wang et al., 2018).

#### 3.5.2 Carbonyl content

In aquatic products, the content of carbonyl compounds is an important indicator for evaluating oxidation levels (Grintzalis et al., 2013). The fresh large yellow croaker samples had a carbonyl content of 1.39 μmol/g pro (Fig. 5B). During cold storage, the carbonyl content in all samples increased to varying degrees, mainly due to the loss of free water leading to reduced water activity and more effective collisions between reactants, accelerating oxidation by iron and other oxidants (T. Li et al., 2014). Additionally, damage to the ultrastructure of muscle cells during cold storage accelerated the release of iron and other oxidants, which might also promote carbonyl production. The carbonyl content in the US + CMCS group was the lowest in the later stages of cold storage, indicating that other treatment groups were less effective in protecting the protein structure, resulting in more severe protein damage in their samples. The interaction of the physical and chemical effects of ultrasound with the bioactive coating led to carbonyl degradation, thus lowering carbonyl content.

#### 3.5.3 Ca^2+^-ATPase

Reduced protein content leads to increased enzyme activity, and Ca^2+^-ATPase activity is measured to evaluate myosin integrity. The loss of Ca^2+^-ATPase activity is attributed to conformational changes and aggregation of the myosin head and increased ionic strength of the system (Chen et al., 2022). Additionally, protein rearrangement through protein interactions also induces the loss of Ca^2+^-ATPase activity. Lower Ca^2+^-ATPase activity indicates less stable protein properties (W. Gao et al., 2019). As shown in Fig. 5C, the Ca^2+^-ATPase of large yellow croaker decreased with storage time, possibly due to oxidation of the myosin head sulfhydryl groups and myosin aggregation (Sun, Zhang, et al., 2019). The ultrasound-assisted group showed a faster decline than the CK group, but the US + CMCS group showed the slowest decline. The combination of ultrasound and *Melissa officinalis* L. essential oil could slow down the denaturation of MP, and the bioactive coating reduced the contact between fish and air, thereby slowing down the rate of protein denaturation. This trend was consistent with changes in sulfhydryl content.

#### 3.5.4 MFI

MFI is an indicator of myofibrillar protein integrity, a higher MFI value indicates more severe fragmentation of myofibrils near the *Z*-line (Stadnik & Dolatowski, 2011). MFI is also an important evaluation index for meat tenderness, positively correlated with myofibril fragmentation and tenderness. Myogenic fibers in muscle are one of the major structures that determine the texture and tenderness of meat. During storage, proteases (e.g., calpain) in the muscle gradually degrade key proteins (e.g., myosin, actin, connexin, etc.) in the myogenic fibers, leading to myofibril breakage. This process increases the MFI value. As the myogenic fibers split, the meat becomes looser, thus increasing tenderness. When myogenic fibers degrade, the protein structure in the muscle loosens and the meat becomes chewier and tender. This structural change directly affects the texture of the meat, with higher MFI values indicating more myogenic fiber breaks and more tender meat. According to Fig. 5D, MFI increased for all samples with prolonged storage time, and there were significant differences in MFI values between the CK group and other treatment groups. This might be because, after the death of large yellow croaker, calpains in the muscle are activated, leading to degradation of Z-line associated MP and thus causing muscle fiber fragmentation (Hopkins et al., 2004). The MFI in the US + CMCS group was significantly higher than in other treatment groups, indicating that the combined treatment of ultrasound and *Melissa officinalis* L. essential oil positively impacted MFI of large yellow croaker. The cavitation effect of ultrasound disrupted the myofibrils and connective tissue in the fillets, accelerating the fragmentation of myofibrils. Furthermore, the cavitation and mechanical effects could produce intense shear effects in a short time, severely damaging MP. Additionally, ultrasound promoted the penetration of *Melissa officinalis* L. essential oil, leading to the weakening and breaking of myofibrillar structure, significantly improving the tenderness of large yellow croaker.

## 4 Conclusion

In this study, the effects of orthogonal dual-frequency ultrasound-assisted treatment combined with *Melissa officinalis* L. essential oil-carboxymethyl chitosan-locust bean gum bioactive coating on the quality, lipid, and MP of large yellow croaker during cold storage were discussed. Analysis of microorganism, TVB-N, and K value indicated that the shelf life of large yellow croaker for CK, US, CMCS, and US+CMCS groups were 6 d, 12 d, 12 d, and 18 d, respectively. LF-NMR and WHC collectively demonstrated that the combination treatment of US+CMCS had the best moisture retention effect. SEM revealed the microstructural impacts of different treatments on the refrigerated large yellow croaker, showing that the combination treatment caused the least structural damage. Ultrasound-assisted, combined with preservatives, significantly enhanced the preservation effect on fish. Furthermore, the experimental results indicated that the combination treatment could delay the oxidative degradation of lipids and MP in large yellow croaker during cold storage. The combination treatment not only maintained the better quality and structure of large yellow croaker but also achieved optimal preservation effects, thus it could be considered an alternative treatment method for refrigerated large yellow croaker. Combining ultrasound technology with essential oil coatings for fish preservation is an innovative preservation strategy that can utilize the synergistic effect of both to delay spoilage and freshness loss of fish. This technology has multiple potentials for practical application, but there are some limitations. Essential oil coatings can be applied in a variety of ways, either by spraying, soaking or applying to form a uniform coating on the surface of aquatic products. The preparation of essential oils into nanoemulsions or composite films is recommended for future research and application to improve their adhesion and permeability on the surface of aquatic products. Combined with ultrasound treatment, essential oils can penetrate into the tissues of aquatic products more effectively to exert antimicrobial and preservation effects. In practice, it is critical to ensure that essential oil coatings and ultrasound treatments are not harmful to humans. Although essential oils are considered natural and safe food additives, certain essential oils may affect the flavor of food or trigger allergic reactions at high concentrations. Therefore, it is recommended to control the amount of essential oils used to ensure compliance with food safety standards while maintaining the flavor and texture of aquatic products unaffected. Consumer acceptance of essential oils and sonication also needs to be considered in the promotion process.

## Funding declaration

This work was supported by the 10.13039/501100012166National Key Research and Development Program of China (2023YFD2401402), the earmarked fund for CARS-47, Shanghai Municipal Science and Technology Project to Enhance the Capabilities of the Platform (20DZ2292200, 19DZ2284000).

## CRediT authorship contribution statement

**Hao Cheng:** Writing – original draft, Data curation. **Chenchen Zhang:** Methodology, Investigation, Formal analysis. **Jinfeng Wang:** Validation, Supervision. **Jing Xie:** Writing – review & editing, Project administration, Funding acquisition.

## Declaration of competing interest

The authors declare that they have no known competing financial interests or personal relationships that could have appeared to influence the work reported in this paper.

## Data Availability

The authors do not have permission to share data.
